# The effects of an educational intervention based on the protection motivation theory on the attitude of mothers regarding the prevention of child poisoning: a quasi-experimental study

**DOI:** 10.1186/s12889-026-26467-5

**Published:** 2026-02-09

**Authors:** Samin Bakhshalizade Rashti, Fatemeh Pashaei Sabet, Saeed Ghasemi, Mahsa Matbouei, Parvin Sarbakhsh

**Affiliations:** 1 Department of Community Health Nursing, Student Research Committee, School of Nursing and Midwifery, Shahid Beheshti University of Medical Sciences, Tehran, Iran; 2 Department of Community Health Nursing, School of Nursing and Midwifery, Shahid Beheshti University of Medical Sciences, Tehran, Iran; 3 Health and Environment Research center, Tabriz University of Medical Sciences, Tabriz, Iran

**Keywords:** Education, Protection motivation theory, Prevention, Child poisoning, Children 1 to 6 years old

## Abstract

**Introduction:**

childhood poisoning is considered a major health problems. In order to prevent child poisoning, providing general education to society may be effective in reducing these types of childhood accidents. Therefore, this study aimed to evaluate the effect of an educational intervention on the attitude of mothers regarding childhood poisoning prevention in Tehran, Iran, using the protection motivation theory.

**Method:**

This study was of a quasi-experimental design. 129 mothers with children aged 1 to 6 years who were referred to selected comprehensive urban health service centers in the city of Tehran were divided into two intervention and control groups by cluster-random sampling method. 64 people were in the intervention group and 65 people were in the control group. Willingness to participate in the study, having at least one child between 1 and 6 years old, having a smart cell phone, mother’s literacy, having a health record in the selected centers were the study inclusion criteria; and on the other hand, not completing answer to the questionnaire, leaving the study at any time, not participating in at least one training session were the exclusion criteria in this study. Educational content based on protection motivation theory was implemented for mothers in the form of two 10-15-minutes educational video sessions and one discussion session virtually. Questionnaires were completed by mothers before, immediately after and one month after the educational intervention. Data analysis was done using SPSS version 23 software in two sections, descriptive statistics and inferential statistics.

**Results:**

The intervention and control groups in terms of demographic characteristics were the same. The mean score of perceived sensitivity and self-efficacy constructs after the educational intervention was significantly higher in the intervention group rather than in the control group (*p* < 0.05). Also, the difference in the scores of other protection motivation theory constructs (perceived severity, fear, response efficacy and response cost) in the two groups was not statistically significant.

**Conclusion:**

The results of this study showed the effectiveness of the educational intervention based on the protection motivation theory on the attitude of mothers with 1-6-year-old children against child poisoning. Therefore, using this theory for making educational programs in health treatment centers could be useful to change the performance of mothers in preventing of poisoning in children at home.

## Background

Unwanted injuries in childhood are one of the main causes of disability and death worldwide; in the United States, unintentional accidents at home have been reported as one of the main causes of death for children over one year of age [1, 2]. Among these accidents in childhood, poisonings are the fourth most common cause of admission of children to the emergency department [3]. Poisonings occur following ingestion, injection, inhalation and skin contact with harmful substances [2, 4] and since children spend most of their time at home, one of the potential risks threatening them is access to toxic substances in the house [5]. According to reports published by UNICEF, more than one million children under the age of five die every year due to the poisoning [6]. In Iran, accidental poisoning of children in recent years have been estimated between 20,000 and 25,000 cases per year [7].

Incidence of poisoning occurs mostly in children under 6 years old [4, 8–10]. An integral part of the cognitive development of children at this age is their desire to search and discover their surroundings; especially, this exploration is done by taking the material to their mouth; on the other hand, their ignorance of the dangers of the medicine makes them susceptible to injury [5]. The developing of the neurocognitive system, imitating the adult’s behaviors, sense of curiosity, lack of adequate supervision by caregivers and easy access to these dangerous substances are other causes of poisoning at these ages [2, 5, 11]. The socio-economic status of the family is also one of the other factors affecting these accidents; According to UNICEF reports, the occurrence of these incidents in children who live in low-income countries is eight times more than regarding the children living in high-income countries [1, 6].

Children’s poisoning is one of the most preventable accidents that can significantly reduce the death rate caused by accidents in children [10]. On the other hand, reducing the number of accidents is considered a global health goal, and the common point in all these accidents is that they can be prevented [12]. The first line of defense to prevent this type of accidents are parents and caregivers, and their behaviors are influenced by their attitudes and awareness towards the proper storage of harmful substances; as many parents think that supervision alone will protect the child from poisoning [13]. For this reason, some measures should be implemented; such as general education for the society, safety warnings along with the packaging of medicine and chemicals, and using suitable containers for storing drugs medicine [14].

One of the theories that is widely used in predicting and intervening in protective behaviors against childhood incidents is the protection motivation theory [15, 16]. The PMT (Protection Motivation Theory) was proposed by Rogers in 1975 to explain the effects of stress and health behaviors and attitudes [13]. The PMT is one of the most efficient theories, which emphasizes the attitude and development of adaptive skills [17]. This theory can be used to investigate the factors affecting the attitude and then to change the person’s behavior [18]. According to this theory, the behaviors related to a person’s health follow the constructs of perceived sensitivity, perceived severity, maladaptive response rewards, fear, self-efficacy, response efficiency, and response costs [19, 20].

In a study conducted on rural children in India, it was found that most poisonings occurred in children under 5 years old; and poisoning with kerosene and acetaminophen tablets were among the most common cases of poisoning [21]. In another study it was found that considering the effective role of mothers in the field of preventing domestic accidents and accidents in children under 6 years old, implementing educational interventions based on the structures of PMT can play a decisive role in reducing domestic injuries in this age group [15].

Since this theory focuses on effective cognitive factors in decision-making when faced with traumatic events, and considering its effective role in various studies, in identifying effective cognitive factors in accident prevention behaviors and promoting these behaviors, so, this study aims to evaluate the effects of an educational intervention based on the protection motivation theory on the attitude of mothers regarding the prevention of child poisoning in 2024.

## Method

### Research design and setting

This quasi-experimental study was conducted on mothers with children aged 1 to 6 years who were referred to four urban health comprehensive services centers. They were divided into two centers for the intervention group and two centers for the control group in Tehran, Iran. The Iranian healthcare system comprises public and private healthcare providers that public sector includes government-funded hospitals, clinics, and health comprehensive services centers. The urban health comprehensive services center is a facility located in urban areas, serving an average population of approximately 12,000 individuals. The primary objective of the urban health comprehensive services center is to provide essential healthcare services to the covered population and, when necessary, facilitate patient referrals to hospitals. Services provided by urban health centers include vaccination, oral health, environmental and occupational health, nutritional consultation, psychologist consultation, outpatient services and if necessary referral system. The intervention was crafted around the principles of Protection Motivation Theory (PMT). Insights gained from an initial literature review and casual discussions with mothers at the participating centers revealed shortcomings in their perceived vulnerability and confidence in preventing poisoning incidents. Taking these observations into account, the educational materials were structured to focus on raising awareness about the dangers of childhood poisoning while providing actionable prevention strategies, ensuring the program was well-suited to address the needs of the intended audience. The study was conducted from February to July 2024.

### Sample size and sampling methods

The sample size was calculated through the Pocock’s formula and according to the similar study [12], taking into account the first type error of 0.05 and the test power of 90%, 43 samples were calculated in each group, and finally, taking into account the 20% probability of dropping, 65 samples were calculated. It was calculated in each group total of 129 people in two groups. Finally, 64 people were in the intervention group and 65 people were in the control group.

This study employed a multistage cluster-random sampling method. In the first stage, four comprehensive urban health service centers were chosen from a pool of 89 centers in Tehran, based on factors such as accessibility, the volume of referrals, and available facilities. Each selected center represented a cluster. To reduce the likelihood of contamination between groups, randomization was performed at the cluster level, assigning two centers to the intervention group and two to the control group. In the second stage, participants were selected within each center using a random number table applied to the list of children aged 1–6 years recorded in the SIB system, Iran’s Integrated Health System, which serves as a national electronic health record database managed by the Ministry of Health. The mothers of these children were then contacted by phone, informed about the study’s goals and procedures, and invited to participate if they expressed willingness.

### Inclusion and exclusion criteria

The study inclusion criteria were willingness to participate in the study, having at least one child between 1 and 6 years old, having a smart cell phone in the family, having literacy for mothers having a health record in the selected centers [12, 22]. The exclusion criteria were incomplete questionnaire responses, withdrawal from the study at any stage, and failure to attend at least one training session.

### Intervention group procedure

**Fig. 1:**
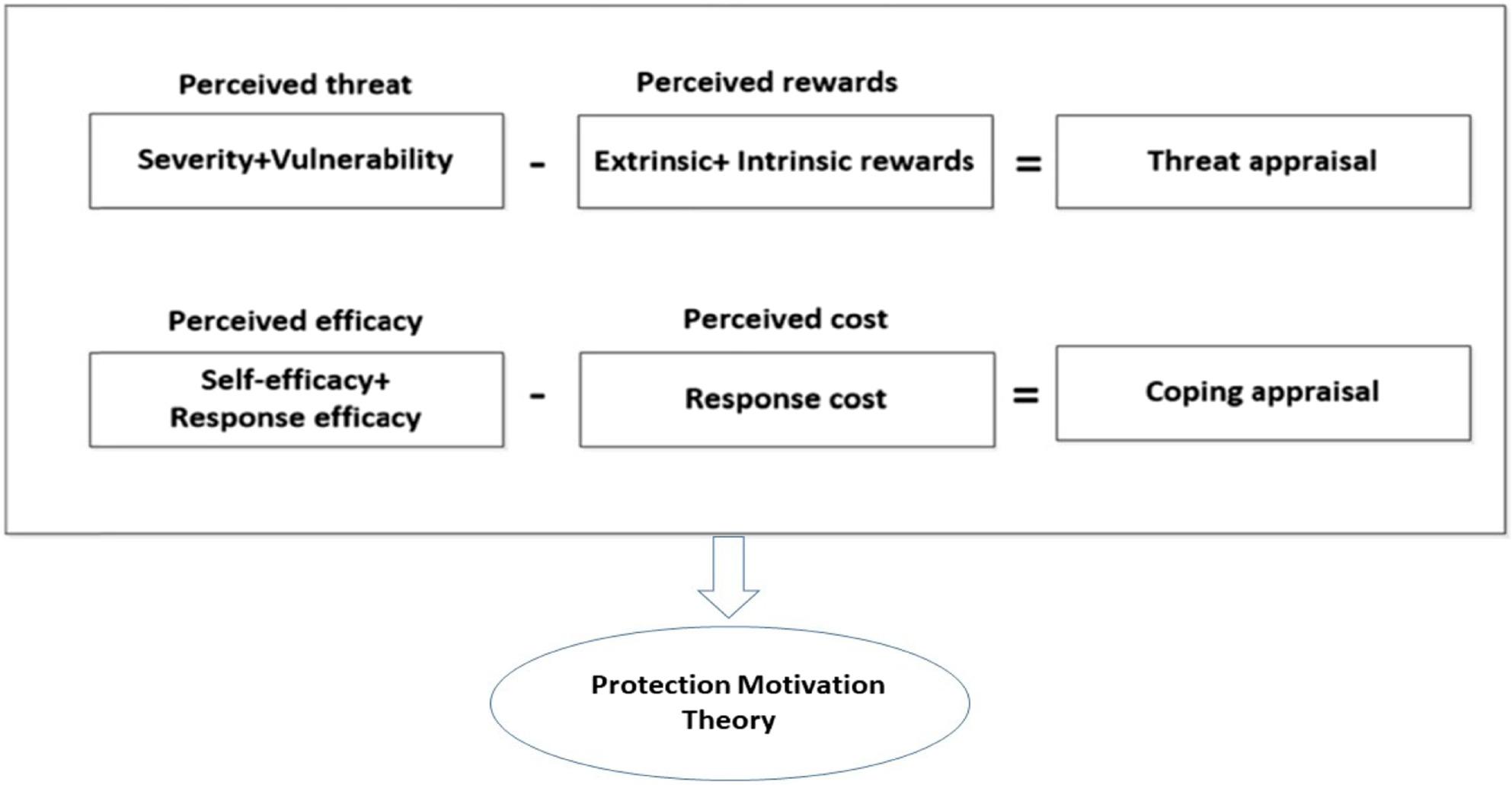
Protection motivation theory framework

After the approval of the proposal, the ethics license with the code IR.SBMU.PHARMACY.REC.1402.19 was issued in the ethics committee. First, the internal reliability of the questions was determined by Cronbach’s alpha method. For this purpose, each construct was completed by 20 mothers who had the same conditions as the samples. The educational intervention included 3 educational sessions in the form of educational videos and group discussion method in the online educational space for the intervention group (Table 1). The content of the educational interventions was designed based on the theory of protective motivation (Fig. 1). To prepare the educational content, scientific texts on home safety training against poisoning in children and the initial symptoms of poisoning and the initial measures in case, were used, and it was reviewed and approved by the supervisor; Then, the educational content was given to 3 nursing professors who were members of the university’s academic faculty to confirm validity. Expressing the contents in simple language, not using specialized words and prioritizing the key points presented in each training session were observed. In order to provide a comfortable accessible environment for mothers, educational videos, group discussions, virtual question and answer sessions and clear communication techniques were used.

**Table 1:** Educational sessions and educational content provided to the participants of the intervention group

Sessions and Time required	Educational goals	Educational content based on PMT constructs	Training methods and materials	Instructors
1 (30–40 min)	Stimulation of threat appraisal constructs (perceived sensitivity, perceived severity and fear)	Introduction and statement of the objectives of the first session, familiarization with the topic of poisoning in children and its importance, familiarization with the types of substances in the home that cause poisoning in children, the ways of poisoning, the statistics of poisoning in children, the importance and causes of poisoning at the age of 1 to 6 years, the complications and consequences of poisoning in children, the importance of securing the home and stating the topic and the time of sending the educational site of the second session	Educational videos uploaded on the designed educational site, group discussion sessions in virtual space on WhatsApp and Telegram applications, PDF training sessions and pamphlets	First author
2 (40–45 min)	Stimulation of coping appraisal constructs (self-efficacy, response cost and response efficacy)	Explaining how to secure the home against poisoning; Among the necessary measures for the safe storage of dangerous chemicals in the home (detergents, cosmetics, poisons and insecticides), medicines, plants. Ways to prevent poisoning with carbon monoxide gas, stating the symptoms of poisoning with each of the mentioned items and basic necessary measures for the occurrence of poisoning in a child.		
3 (45–60 min)	Summarizing and reviewing the contents	Summarizing the presented content and discussing in the virtual space about the solutions provided against poisoning and the importance of this issue and sharing the mothers’ points of view on this matter in the group.		

To determine the effects of the educational intervention and to compare the results between the groups, immediately and one month after the last training session in the intervention group, the questionnaires were again provided to the research samples for completion.

### Control group procedure

**Fig. 2:**
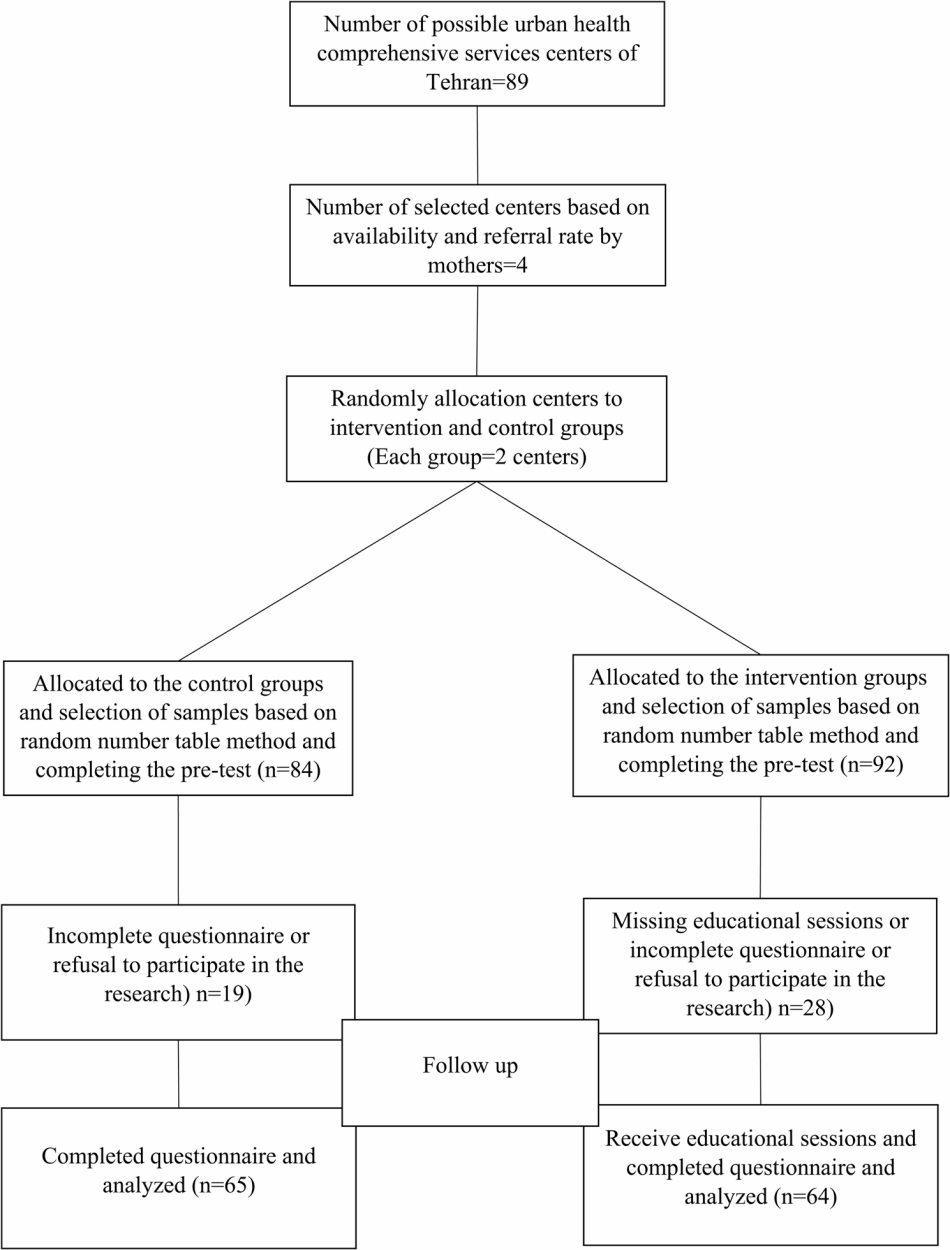
Consort diagram

The control group completed the questionnaires at the same time as the intervention group in 3 time periods (before, immediately after and one month after the intervention) with the difference that they did not receive any training during these periods. After collecting the last series of questionnaires, one month after the educational intervention, educational content in the form of videos, PDFs and pamphlets were sent for the cell phones of all mothers in the control group (Fig. 2).

### Instruments

The tool used for data collection included two parts; Demographic information form (8 items) and protection motivation theory construction measurement scale (31 items).

The demographic information form included mother’s age, mother’s occupation, mother’s education, father’s education, father’s occupation, number of children, gender of 1 to 6-year-old child, and history of child poisoning in the past.

The scale measuring the constructs of the theory of protection motivation includes the items of perceived sensitivity (items 1–7), perceived severity (items 8–10), maladaptive response rewards (items 11 and 12), fear (items 13–15), self-efficacy (items 16–23), response cost (items 24–27) and response efficacy (items 28–31).

This questionnaire was used in Rahimi et al.‘s study in 2014 [22]. In order to review the questionnaire and adapt it to the goals of the present study, the items were reviewed through the review of the related texts and with the opinion of toolmaking experts, as a result, 10 items were removed and psychometric evaluations were conducted.

Face validity was assessed by giving the questionnaire to 20 mothers who had inclusion criteria but were not part of the main sample. They were asked to evaluate the appropriateness, clarity, ambiguity, and complexity of the items [23, 24].

Content validity was evaluated using both qualitative and quantitative approaches. Three experts in community health education and tool development reviewed the questionnaire for necessity, relevance, placement of items, sufficiency, grammar, and use of simple words. Based on their input, the content validity ratio (CVR) for the entire questionnaire was 0.80 and the content validity index (CVI) was 0.79 [22, 25].

Reliability was examined using Cronbach’s alpha method in the current study population. The questionnaire was given to 20 mothers with similar characteristics to the target group, and Cronbach’s alpha coefficients for perceived sensitivity, self-efficacy, response cost, and response efficacy were 0.786, 0.664, 0.732, and 0.771, respectively. The constructs of fear and maladaptive response rewards were single-question inconsistent responses; therefore, Cronbach’s alpha could not be calculated for them.

### Data analysis

Descriptive statistical indices including mean and standard deviation for quantitative variables and number and percentage for qualitative variables and analytical tests including chi-square test, Fisher’s exact test and variance analysis test with repeated measurements were used to describe and analyze the data. Variance analysis tests with repeated measurements and independent t-test were used to investigate intra-group (time effect) and inter-group changes in intervention and control groups of mothers participating in the study according to the constructs of PMT. In order to check the normality of the data, skewness and kurtosis indices were calculated and reported. Analyzes were performed at a significant level (*p* < 0.05) and two-way with SPSS 23 software.

## Results

There were 129 participants in this study, 64 in the intervention group and 65 in the control group. The mean age of mothers in the intervention group was (33.27 ± 6.85) and in the control group was (33.63 ± 5.17). Most of the mothers were housewives and had a bachelor’s degree. The intervention and control group participants in terms of eight variables (mother’s employment status, father’s employment status, mother’s educational level, father’s educational level, child’s gender, history of child’s poisoning in the past, mother’s age and the number of children in the family) were the same (Table 2).

**Table 2:** Demographic characteristics of the intervention and control groups

Demographic variables	Categories	Intervention *n*(%)	Control *n*(%)	*p*-value
Employment status (mother)	Housewife	46(71.9)	51(78.5)	0.386
	Employed	18(28.1)	14(21.5)	
Employment status (father)	Employed	64(100)	62(95.4)	0.244
	Without a job	0(0.0)	3(4.6)	
Level of Education (mother)	Doctorate	1(1.6)	1(1.5)	0.314
	Master of Science	14(21.9)	8(12.3)	
	Bachelor of Science	29(45.3)	26(40.0)	
	Diploma	16(25.0)	27(41.5)	
	High school	4(6.3)	3(4.6)	
Level of Education (father)	Doctorate	2(3.1)	2(3.1)	0.120
	Master of Science	14(21.9)	6(9.2)	
	Bachelor of Science	28(43.8)	23(35.4)	
	Diploma	15(23.4)	25(38.5)	
	High school	5(7.8)	9(13.8)	
Child gender 1 to 6 years	Girl	32(50)	34(52.3)	0.122
	Boy	28(43.8)	31(47.7)	
History of child poisoning in the past	Yes	4(6.3)	4(6.2)	1.000
	No	60(93.8)	61(93.8)	
Age (mother)	Years	33.27 ± 6.85 ^a^	33.63 ± 5.17 ^a^	0.833
Number of children	Number	1.47 ± 0.67 ^a^	1.68 ± 0.82 ^a^	0.244

The results of the independent samples t-test showed that before the intervention, there was no statistically significant difference between the mean scores of all PMT constructs between the intervention and control groups (*p* > 0.05). Immediately after the intervention, the mean scores of maladaptive response rewards and self-efficacy constructs were significantly higher in the intervention group rather than in the control group, and the mean scores of the response efficacy construct in the control group were significantly higher than the intervention group (*p* < 0.05). One month after the intervention, only the mean scores of perceived sensitivity, maladaptive response rewards, fear and self-efficacy in the intervention group were significantly higher than the control group (*p* < 0.05) (Table 3).

**Table 3:** Comparison of the total and sub-scales mean score of mothers’ attitude against child poisoning in the intervention and control groups over time

Mothers’ attitude	Time point	Group		t(*p*-value)
		Intervention	Control	
		Mean (SD)	Mean (SD)	
Perceived sensitivity	Pre-intervention	3.43(1.05)	3.34(0.97)	0.469 (0.640)
	Immediately after intervention	3.33(0.77)	3.25(0.93)	0.515 (0.607)
	One-month after intervention	3.71(0.57)	3.17(0.91)	3.824 (<0.001)^*^
F (*p*-value)		5.244 (0.013) ^*^	12.556 (<0.001) *	
Interaction of time and group by mixed ANOVAF (*p*-value)		8.907(0.001) ^*^		
Perceived severity	Pre-intervention	4.26(0.61)	4.11(0.79)	1.194 (0.235)
	Immediately after intervention	3.61(0.67)	3.84(0.69)	-1.837 (0.069)
	One-month after intervention	3.64(0.58)	3.69(0.59)	-0.497 (0.620)
F (*p*-value)		22.529 (<0.001) *	24.802 (<0.001) *	
Interaction of time and group by mixed ANOVAF (*p*-value)		3.34(0.049) ^*^		
Maladaptive response rewards	Pre-intervention	2.23(1.25)	2.35(1.19)	-0.554 (0.580)
	Immediately after intervention	3.06(1.08)	2.51(1.16)	2.691 (0.008) ^*^
	One-month after intervention	3.35(1.06)	2.68(1.11)	3.314 (0.001) ^*^
F (*p*-value)		25.124 (<0.001) *	7.118 (0.002) ^*^	
Interaction of time and group by mixed ANOVAF (*p*-value)		8.895(0.001) ^*^		
Fear	Pre-intervention	4.50(0.69)	4.30(0.80)	1.452 (0.149)
	Immediately after intervention	4.15(0.78)	4.03(0.74)	0.865 (0.389)
	One-month after intervention	4.32(0.65)	3.96(0.87)	2.493 (0.014) ^*^
F (*p*-value)		4.994 (0.013) ^*^	4.712 (0.017) ^*^	
Interaction of time and group by mixed ANOVAF (*p*-value)		1.172(0.309)		
Self-efficacy	Pre-intervention	4.20(0.47)	4.05(0.54)	1.671 (0.097)
	Immediately after intervention	4.10(0.44)	3.77(0.39)	4.376 (<0.001) *
	One-month after intervention	4.40(0.39)	3.61(0.33)	11.709 (<0.001) *
F (*p*-value)		8.986 (0.001) ^*^	57.991 (<0.001) *	
Interaction of time and group by mixed ANOVAF (*p*-value)		32.760(<0.001) *		
Response costs	Pre-intervention	2.44(0.81)	2.43(0.90)	0.096 (0.923)
	Immediately after intervention	2.67(0.64)	2.45(0.81)	1.689 (0.094)
	One-month after intervention	2.56(0.67)	2.47(0.64)	0.734 (0.465)
F (*p*-value)		2.827 (0.091)	0.266 (0.696)	
Interaction of time and group by mixed ANOVAF (*p*-value)		1.669(0.198)		
Response efficacy	Pre-intervention	4.23(0.80)	4.29(0.53)	-0.545 (0.587)
	Immediately after intervention	3.93(0.42)	4.15(0.50)	-2.544 (0.012) ^*^
	One-month after intervention	4.21(0.45)	4.20(0.53)	0.054 (0.957)
F (*p*-value)		4.760 (0.026) ^*^	5.412 (0.010) ^*^	
Interaction of time and group by mixed ANOVAF (*p*-value)		1.938(0.162)		

^*^Significant at *p*<0.05

Intragroup comparison using the one-way repeated measure ANOVA (Analysis of Variance) showed that in the intervention group, the constructs of perceived sensitivity, maladaptive response rewards and self-efficacy increased significantly over time; while the constructs of perceived severity, fear and response efficacy were significantly reduced. In the control group, the constructs of perceived sensitivity, perceived severity, fear, self-efficacy and response efficacy decreased significantly over time (*p* < 0.05) (Table 3).

The final effect of the intervention showed that the educational intervention significantly improved the constructs of perceived sensitivity and self-efficacy while it decreased the construct of perceived severity (*p* < 0.05).

## Discussion

The present study aimed to determine the effects of an educational intervention based on the protection motivation theory on the attitude of mothers against child poisoning. According to the findings, mothers in the intervention and control groups were similar in terms of demographic characteristics.

The results of the present study showed that most of the samples in both intervention and control groups were housewives with a diploma or higher education and they were similar in terms of employment status. The mean age of the participants in the intervention group was 33.27 ± 6.85 and in the control group was 33.63 ± 5.17. Most of the mothers in both intervention and control groups mentioned that there was no history of poisoning in their children. Also, most of the fathers in the intervention and control groups were employed and had a diploma or higher education. The results showed that one month after the educational intervention, the mean score of perceived sensitivity in the intervention group was significantly higher than the control group. the mean scores of the perceived sensitivity in the intervention group, generally increased; in other words, the educational intervention was effective in the construct of perceived sensitivity. If the mother perceives that by exposing her child to poison, she or he may be harmed and threatened, she is more likely to perform protective behaviors [17]. This result was consistent with the findings a study entitled “Effect of educational intervention based on protective motivation theory on preventive behaviors of respiratory infections of hospital employees” [26]. But it was not in line with the findings of a study on improving nutritional behaviors and physical activity in military patients with type 2 diabetes, in this study, there was no significant change in the consrtuct of perceived sensitivity after the intervention [27].

Regarding the construct of perceived severity, there was no significant statistical difference between the two intervention and control groups in the matter of time, including before, immediately after and one month after the intervention; accordingly the mean scores of this construct decreased significantly in both groups over time, This fact can indicate the limited awareness of mothers in the field of the consequences of poisoning and the inadequacy of the intervention period to change their attitudes. Using the experiences of people with a history in the field of child poisoning during educational intervention can improve the level of perceived severity in mothers. The results of the present study were consistent with a study in the field of cervical cancer prevention in Iranian women [28]. This result was not in line with the findings of the study regarding the promotion of preventive behaviors against Covid-19 [29]. This contradiction can be due to the high perceived threat of the Covid-19 disease during the epidemic of this disease due to the bombardment of information and conflicting news that was created in the society regarding this disease.

The mean of the maladaptive response rewards construct increased significantly in the intervention group and during the time points, and this increase was also significant in the control group. In general, the lower the amount of reward for incompatible behavior (non-protective behavior), the more likely protective behavior is performed [29]. According to the findings of the present study, the educational intervention increased the perceived reward component, which could be due to the insufficient number of questions related to this construct in the questionnaire. This results were consistent with a study entitled “Effect of training based on protective motivation theory on improving protective behaviors of medical laboratory workers in Yazd, Iran“ [30]. Also, the study regarding the promotion of preventive behaviors of pediculosis in elementary school girls did not evaluate the perceived reward component [31].

The mean scores of the fear construct in both intervention and control groups decreased significantly during the time points. In a study in Iran, related to the effect of an educational intervention based on the theory of protection motivation on the prevention of cervical cancer, there was no significant difference in terms of the mean fear score after the intervention [29], the results of which were consistent with the present study. The results of two studies regarding the effect of educational intervention based on this theory showed a significant increase in the fear construct between the intervention and control groups [26, 32]. , which was not in line with the results of the present study, This discrepancy could be due to the difference in the number of sessions and educational methods in studies.

The mean scores of the self-efficacy construct immediately after and one month after the educational intervention in the intervention group were significantly higher than the control group. Evidence based on the protection motivation theory shows that the purpose of the educational intervention was to increase self-efficacy in mothers with 1–6 year old children; in other words, in this study, the educational intervention based on theory was effective in the self-efficacy construct. In fact, when a person does not have the necessary self-efficacy, he or she cannot perform preventive behaviors, and as a result, they are exposed to harm [26]. These results were in line with the findings of two studies in Iran, regarding the effect of educational intervention based on the theory of protection motivation on the intention to perform a Papanicolaou test [12, 28].

The mean scores of response cost construct were not significantly different between the intervention and control groups at any of time points. The lower the costs of the maladaptive response, the greater the individual’s motivation to create health-related behavior [33]. In one study, similar to our study, no significant effect was observed in the response cost component after the educational intervention [30].

There was no statistically significant difference between the mean scores of the construct of response efficacy of the intervention and control groups, before and one month after the educational intervention the results of one study in Iran, showed that after the educational intervention, all constructs of the theory of protective motivation except response efficacy construct increased significantly [34]. In another study, there was no significant difference between the response efficacy construct in the intervention group and after the educational intervention [30], and the results of these studies were consistent with the present study.

Recent studies highlight the effectiveness of parent-centered education in preventing unintentional childhood injuries. A pilot study in Japan demonstrated that home-visiting parent training programs (SafeCare) improved parents’ knowledge and practices in promoting child safety [35]. A systematic review also reported that pediatric home safety programs for parents effectively enhance safety behaviors and prevent injuries, including poisoning [36]. Additionally, a scoping review of interventions for preventing unintentional injuries emphasized the overall value of parent-focused programs in reducing childhood hazards [37]. Although most studies did not directly assess Protection Motivation Theory (PMT) constructs, they emphasize the importance of parent education in promoting protective practices. Our study builds on this by explicitly applying PMT, filling a gap in the literature, and showing its value in improving mothers’ protective behaviors at home.

### Limitation

There are several limitations which could be considered in this study. Cronbach’s alpha for the perceived severity construct was low, so the results of the perceived severity construct were interpreted and reported with caution. It was not possible to directly observe the behavior of the participants in this research, and the results were limited to the reports of the behavior by the participants. The statistical population of the current research is another limitation of this study that limits the generalizability of the results; therefore, it is suggested to conduct similar studies on other child caregivers, including fathers, older siblings, and child nurses at home. The number of training sessions was another limitation of this study; it is suggested to conduct more training sessions in future studies. Also measuring the behavior after training based on the protection motivation theory is recommended.

## Conclusion

Since behavior is a complex process and changing it is not easy, nevertheless, the present study, by holding three training sessions and collecting data after one month from the last training session, showed that training with emphasis on the protection motivation theory constructs made changes in the attitude of mothers in order to promote child poisoning preventive behaviors at home. Therefore, the use of this theory in formulating educational programs in health-treatment centers to change the performance of mothers in the prevention of children poisoning children at home will promise beneficial effects.

## Data Availability

The datasets used and/or analyzed during the current study are available from the corresponding author on reasonable request.

## Abbreviations

PMT: protection motivation theory
SIB: Electronic health records (EHRs) were introduced in Iran in 2014 with the launch of the Integrated Electronic Health System (SIB)
ANOVA: Analysis of Variance
MD: Mean Difference
SD: Standard Deviation
